# Close Ties between the Nuclear Envelope and Mammalian Telomeres: Give Me Shelter

**DOI:** 10.3390/genes14040775

**Published:** 2023-03-23

**Authors:** Gaëlle Pennarun, Julien Picotto, Pascale Bertrand

**Affiliations:** 1 Université Paris Cité, INSERM, CEA, Stabilité Génétique Cellules Souches et Radiations, LREV/iRCM/IBFJ, F-92260 Fontenay-aux-Roses, France; 2 Université Paris-Saclay, INSERM, CEA, Stabilité Génétique Cellules Souches et Radiations, LREV/iRCM/IBFJ, F-92260 Fontenay-aux-Roses, France

**Keywords:** telomere maintenance, shelterin, nuclear envelope, lamins, DNA repair, genome stability, alternative lengthening of telomeres, nuclear pore

## Abstract

The nuclear envelope (NE) in eukaryotic cells is essential to provide a protective compartment for the genome. Beside its role in connecting the nucleus with the cytoplasm, the NE has numerous important functions including chromatin organization, DNA replication and repair. NE alterations have been linked to different human diseases, such as laminopathies, and are a hallmark of cancer cells. Telomeres, the ends of eukaryotic chromosomes, are crucial for preserving genome stability. Their maintenance involves specific telomeric proteins, repair proteins and several additional factors, including NE proteins. Links between telomere maintenance and the NE have been well established in yeast, in which telomere tethering to the NE is critical for their preservation and beyond. For a long time, in mammalian cells, except during meiosis, telomeres were thought to be randomly localized throughout the nucleus, but recent advances have uncovered close ties between mammalian telomeres and the NE that play important roles for maintaining genome integrity. In this review, we will summarize these connections, with a special focus on telomere dynamics and the nuclear lamina, one of the main NE components, and discuss the evolutionary conservation of these mechanisms.

## 1. Introduction: Nuclear Envelope and Telomere Maintenance in Mammals

### 1.1. The Nuclear Envelope

**Figure 1:**
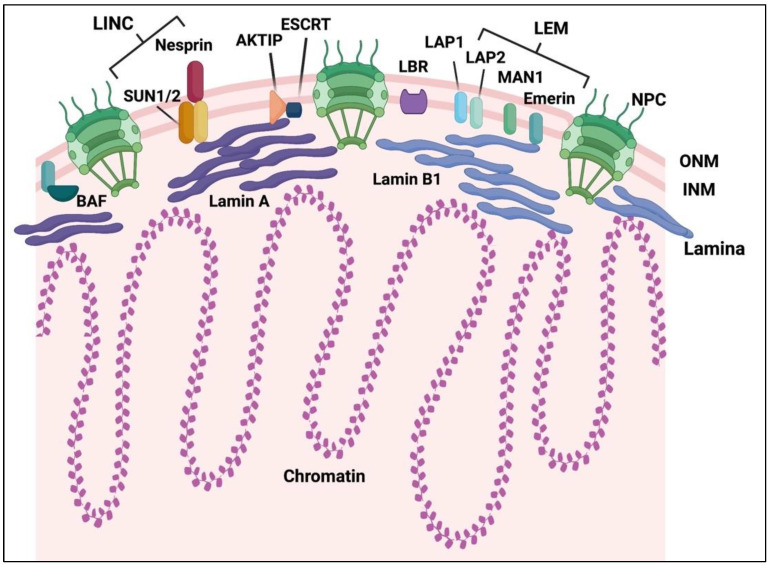
Nuclear envelope in mammals. Graphical representation of the mammalian nuclear envelope (NE) and its key components. The NE is composed of two layers, the inner and outer nuclear membrane (INM and ONM), the nuclear pore complex (NPC) and the nuclear lamina network connected with chromatin. Several key components of the NE are shown: the Linker of the Nucleoskeleton and Cytoskeleton (LINC) complex (SUN1/2, and nesprin proteins), the main lamins (lamin A and B), LAP1, the LEM family (LAP2, Emerin and MAN1), the lamin B receptor (LBR), as well as BAF, which interacts with both LEM and lamina, AKT Interacting Protein (AKTIP) in connection with lamins and the Endosomal Sorting Complex Required for Transport (ESCRT) complex.

Eukaryotic nuclei are bound by a double membrane that plays crucial roles that extend well beyond the preservation of genome integrity. In mammals, this nuclear envelope is principally structurally composed of the outer nuclear membrane (ONM), which is continuous with the endoplasmic reticulum and in direct contact with the cytoplasm, the inner nuclear membrane (INM) that surrounds the nucleoplasm, the nuclear pore complexes (NPCs) and the nuclear lamina (NL) [1] (Figure 1). The NE faces important challenges. While the NE is primordial for protecting the genome and confering the shape and size of the nucleus, it is also a highly dynamic structure. Indeed, at each cell division, the nucleus has to disassemble and reassemble during mitosis. In addition, the NE, owing to its flexibility and changes in properties (stiffness and viscosity), is able to adapt its shape to a certain extent, during cellular processes such as migration and differentiation [1,2]. NE alterations have been reported during the aging process as well as in several human pathologies including nucleopathies and cancers, highlighting its critical role in genome stability [3,4,5].

The NPCs play a key role in the regulation and control of the entrance and export of selected molecules, proteins and mRNAs into the nucleus [6]. The NPCs are mainly composed of around 30 types of different specific proteins, the nucleoporins, that have been highly conserved during evolution from yeast to human and each present in multiple copies. The structure of the NPC forms a cylindric central channel composed by the core nucleoproteins, which perforates the NE followed by an extension of filaments toward the cytoplasmic side and terminates by a basket structure that extends into the nucleus [6].

The nuclear lamina, found in all metazoans, forms a fibrous network under the INM [7]. The lamina is composed of lamins, type V intermediate filaments, which are crucial to maintain the nuclear architecture and beyond. Indeed, they play additional roles in various cellular processes including DNA replication and repair, chromatin organization and mitosis. Lamins are involved in the anchoring of large genomic regions at the nuclear lamina, called Lamina-associated domains (LADs). These LADs are mainly composed of heterochromatin associated with transcriptionally silent regions. Mammal organisms express two types of lamins, A- and B-types, that form separate networks, although these networks are interconnected [8,9]. Of note, a pool of lamins is also present in the nucleoplasm, outside of the lamina [10]. This pool plays a major role in chromatin organization and was proposed to act as a nucleoplasmic scaffold or attachment matrix and also to be involved in mechanosignaling [10,11]. Among the lamins, lamin B1 is expressed in almost all somatic cells (from stem cells to differentiated tissues) from early embryogenesis and throughout life, while the expression of lamin A is more restricted to differentiated cells and mostly detected after birth [11,12]. Although lamins share common functions such as the maintenance of the NE structure, lamin A and B1 are not interchangeable and also carry out distinct functions. Indeed, in addition to differences in expression pattern and maturation [11], as well as localization in separate meshworks, they confer distinct mechanical properties to the nucleus, in terms of elasticity and viscosity [13], and differentially interact with chromatin [14]. They form networks with common but also distinct protein partners [15]. Furthermore, their genetic alterations give rise to distinct human pathologies [16].

Besides the lamina, one other important component of the NE is the LINC complex, that bridges the NE to the cytoskeleton at the outer face and to the lamina and other INM-associated proteins at the inner face of the NE [17]. In mammals, the LINC complex is composed of four main proteins: SUN1, SUN2, nesprin-1 and nesprin-2. The SUN1 and SUN2 proteins contain a transmembrane domain, a C-terminal part emerging inside the lumen of the NE, and an N-terminal part extending in the nucleus and interacting with lamin A. Nesprin-1 and -2 are localized in the ONM and interact with the actin and microtubule networks through their cytoplasmic N-terminal domains, while through their KASH domain (Klarsicht, ANC-1, Syne homology), they bind SUN proteins [17]. Other INM proteins have been reported to play important roles in the functional and physical integrity of the NE, including the lamin B receptor (LBR), lamina-associated polypeptide 1 (LAP1), as well as the LAP2, Emerin, and MAN1 proteins, forming the LEM-domain family [18]. These LEM proteins directly bind to lamins and to the barrier-to-autointegration factor (BAF), a protein required for nuclear envelope reformation at the end of mitosis. In addition, the factor AKTIP has been reported to interact with lamins at the nuclear rim [19] and to be involved in the Endosomal Sorting Complex Required for Transport (ESCRT) pathway, which is required for the completion of cytokinesis by cleaving the cytokinetic bridge between the two daughter cells [20].

### 1.2. Telomere Maintenance

Telomeres, which constitute the ends of eukaryotic chromosomes, are essential for stabilizing chromosomes and protecting them against degradation and recombination [21]. Telomere maintenance, which involves telomere-specific proteins (shelterin complex, detailed below) and DNA replication and repair proteins, is crucial for cell growth and survival. Alterations of these maintenance mechanisms play an important role in the genesis of cancers but also in premature aging. In mammals, telomeres contain 5–15 kb of double-stranded DNA repeats (5′-TTAGGG-3′) terminated by a G-rich single-stranded 3′ extension. One of the essential functions of telomeres is to act as a mitotic clock. Indeed, with each cell division, the telomeres of somatic cells shorten by 50 to 200 bp, due in part to the inability of DNA polymerase to completely replicate DNA ends, known as the “end-replication problem” [22,23]. In addition, the telomeric DNA ends are further processed by nucleases to restore the 3′ overhangs, contributing to shorten telomeres [24,25,26,27]. When telomeres have shortened below a critical size, due to this progressive telomere shortening with cell division, cells stop dividing and enter senescence, a phenomenon known as replicative senescence. Telomerase, a ribonucleoprotein complex, stabilizes telomere length by adding telomeric repeats through its reverse transcription activity [28]. After birth, telomerase expression is repressed in most human somatic cells although limited expression in the S phase has been observed in normal cycling cells [29], but not sufficiently to maintain the telomere’s length. This enzyme is reactivated in most tumor cells (85–90%) and expressed in germ cells and pluripotent stem cells during development [30]. Activation of telomerase seems necessary for cancer cells to escape senescence and to proliferate abnormally. However, telomere length in some tumors (10–15%), mainly sarcomas and tumors of the central and peripheral nervous system, can be maintained by a telomerase-independent system called the Alternative Lengthening of Telomeres mechanism or ALT [31,32]. This mechanism is based on a form of homology directed repair (HDR) pathway close to Break-induced-replication (BIR) involving exchanges between the telomeres of sister chromatids [33,34]. Common characteristics of ALT activity in cells have been reported [34]: lack of telomerase activity, formation of ALT-associated promyelocytic leukemia (PML) bodies [35], high level of Telomere-sister chromatid exchange (t-SCE) [36], formation of circular extrachromosomal telomere repeats (ECTR or telomeric C-circles) [37] and highly heterogenous telomere length [38]. ALT-associated PML bodies contain aggregates of telomeres with several shelterin, DNA replication and repair proteins, including homologous recombination (HR) factors, and are thought to be the site of telomere elongation via telomere recombination [39].

For many years, telomeres were believed to be silent DNA regions. However, it has been shown that telomeres are transcribed from subtelomeric regions toward chromosome ends into RNA containing telomeric repeats of varying sizes (TERRA) [40]. TERRA RNAs are largely located at the telomere and have been involved in the formation of heterochromatin [41,42], as well as in the maintenance of telomeres, through HDR promotion and telomerase regulation [43,44,45,46].

**Figure 2:**
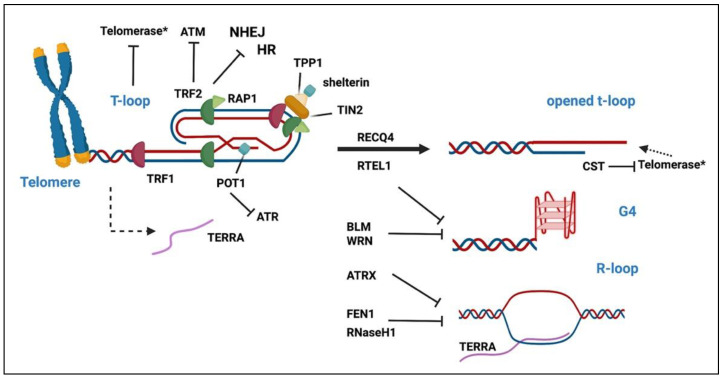
Telomere maintenance in mammals. Graphical representation of the mammalian telomere and the key factors involved in its maintenance. The telomere, the end of chromosome, is composed of a double-stranded DNA part, consisting of TTAGGG repeats in tandem, followed by a single 3′ overhang that invades the telomeric double-stranded DNA. This invasion generates a protective structure, called the t-loop, stabilized by the shelterin complex composed of six telomeric proteins (TRF2, TRF2, RAP1, POT1, TIN2, TPP1). TRF2 has a key function in inhibiting the activation of ATM signaling and inappropriate repair by HR and NHEJ. Once the telomere opens during the S phase, the telomerase, in telomerase-positive cells (*), can bind telomeres and add telomeric repeats. Then, the CST complex inhibits telomerase to prevent excessive telomere extension. Telomeres can also adopt unusual structures, such as G4, due to the G-rich content of the telomeric DNA sequence, or R-loop formed by the association of telomeric DNA with TERRA, the non-coding telomeric transcript of the telomere. These structures must be dismantled by the indicated proteins for replication to continue.

Telomeres are particularly challenged by two major stresses: replication stress and oxidative stress [47]. Given the guanine-rich content of their repeats, telomeres are prompted to form unusual structures, such as G-quadruplex (G4) structures, which must be resolved to allow the completion of telomere replication; in addition, their association with TERRA could lead to the formation of DNA:RNA hybrid structures (R-loops) that could also constitute an obstacle during telomere replication [44]. Moreover, telomeric DNA is highly sensitive to oxidative stress, in part, due to its high content of guanines, and can accumulate oxidative damage with age contributing to enhanced telomere shortening and accelerated cellular senescence [48]. To respond to these major challenges, telomeres possess an elaborate maintenance system involving specific telomere proteins and DNA-damage response proteins, DNA repair and replication proteins, as well as other transient interacting factors discovered in recent years. First, telomeres are capped by a specific protein complex, the so-called “shelterin” complex, which is essential for their protection [49,50,51,52] (Figure 2). The shelterin complex is composed of six main proteins (TRF2, TRF1, POT1, RAP1 TPP1 and TIN2) [53] and interacts with the double-stranded and the single-stranded DNA of the telomere to protect it by giving it a specific t-loop conformation, which consists of the invasion of the G-rich single-stranded 3′ end into the preceding double-stranded telomeric sequence [54]. The existence of this structure has been further confirmed by its observation using super-resolution microscopy [55]. This capped conformation of telomeres prevents them from being recognized as DNA double-strand breaks by the DNA repair machinery [56]. Indeed, deprotection of telomeres elicits a DNA damage-like response, marked by telomere dysfunction-induced foci (TIFs), leading to cell cycle arrest, inappropriate repair, genomic instability, and cell death or senescence depending on the cellular context [57]. Among the shelterin proteins, TRF2, is essential for the stabilization of the t-loop structure. Notably, TRF2 binds telomeric DNA repeats as an homodimer, through its Myb domain and stimulates strand invasion of the 3′ overhang into duplex DNA [58]. TRF2 also directly participates in the inhibition of the ATM-dependent DNA damage response signaling pathway, as well as in the inhibition of non-homologous end joining (NHEJ) and HR repair mechanisms, notably via its interactions with several factors involved in these pathways [59,60,61]. RAP1 is recruited to telomeres via its interaction with TRF2 [62], which enhances the affinity of the latter for telomeric DNA [63]. RAP1, in cooperation with TRF2, is also involved in the protection against NHEJ [64,65,66,67] and in the inhibition of HR [68,69]. Although POT1 directly binds to the telomeric single-strand overhang, its recruitment at the telomere requires its interaction with TPP1. In humans, POT1 plays a role in repressing the ATR signaling pathway at telomeres as well as in telomeric DNA maintenance, whereas in the mouse, two paralogs of POT1 coexist, POT1a and POT1b (derived from a *Pot1* gene duplication event), which, respectively, carry one of these two functions. In mammals, POT1 has been shown to participate in the control of telomeric C-strand processing (via recruitment of the CST complex, see below) [70], and in telomere replication [71]. POT1 is also involved in telomere lengthening through the control of telomerase recruitment and processivity [72,73,74].

In addition to telomeric proteins and TERRA, telomere maintenance requires additional proteins including the single-strand DNA-binding CTC1-STN1-TEN1 (CST) complex, involved in telomerase regulation and C-strand synthesis [52,75]. Several DNA-damage response proteins, and DNA replication and DNA repair proteins, are also present at telomeres. Some of them are involved in telomere protection, such as ATM, ATR or DNA-PK [65,76,77,78,79,80,81], in the completion of telomere replication by facilitating the removing of G4 (such as the helicases BLM, WRN, RTEL1 or ATRX) [82] or R-loops (i.e., ATRX, RNaseH1, FEN1) [44], or by contributing to resolve the t-loop (i.e., RTEL1, RECQL4) [83,84]. Recently, several other additional factors involved in telomere maintenance have been discovered that are transiently recruited at telomeres or interacting with the shelterin. Among them are components of the nuclear envelope, including lamins, LAP proteins and interacting-lamins proteins (i.e., AKTIP, BAF) (see Section 3.2 and Section 3.3).

### 1.3. Connections between the Nuclear Envelope and Telomeres

Links between the nuclear envelope and telomeres have been well established in yeast models in which telomeres aggregate and tether at the NE [85,86]. Relocalization of yeast telomeres to the nuclear periphery is particularly important for telomere replication, transcriptional repression and to protect telomeres from inappropriate recombinations [86,87]. In mammalian germ cells, meiotic telomeres and their connection with the NE have been well investigated in the past years [88]. However, less is known concerning the connections between mammalian telomeres and the NE in somatic cells, as well as in cancer cells, owing to the fact that mammalian telomeres were believed to be randomly localized all over the nucleus and attached to the nuclear matrix, with few of them found at the nuclear periphery. Recent advances emphasize that, as in yeast, mammalian telomeres are also dynamic structures in the nucleus and can transiently relocalize at the nuclear periphery in some instances. In this review, we will discuss in detail the circumstances where repositioning of telomeres occurs at the nuclear periphery, the underlying mechanisms, and the potential roles of the connections between the telomere system and the NE.

## 2. Close Ties between the Nuclear Envelope and Telomere Maintenance: Overview

### 2.1. Telomeres Tethering to the Nuclear Envelope from Yeasts to Mammals

The close proximity between the nuclear envelope and telomeres has been described in several eukaryotic species over the past 40 years, including flies, yeasts, plants and, more recently, mammals. The first observations of telomere anchoring to the nuclear periphery were reported in the seventies, in different species of flies (i.e., *Simulium* or black fly and *Chironomus*). Indeed, the authors observed, via optical microscopy, that most of the telomeres of the polytene chromosomes of these flies are located on the nuclear envelope [89]. In the following years, telomeres were also found to localize close to the nuclear periphery in other organisms. In yeast, telomeres were shown to be not randomly localized throughout the nucleoplasm but preferentially localized at the nuclear periphery [85]. Indeed, in yeast, telomeres form clusters localized at the NE in growing cells of the budding yeast *Saccharomyces cerevisiae* (*S. cerevisiae*) [90,91], as well as in G2 phase cells for the fission yeast *Schizosaccharomyces pombe* (*S. pombe*) [92]. Peripheral telomere localizations in yeast have been reported to play important roles in transcriptional silencing as well as in preventing the telomere from inappropriate recombination [86]. Mechanisms involved in telomere tethering to the NE have been well described in yeast and require the Esc1–Sir4–Rap1 and yKu–Mps3 pathways in *S. cerevisiae* and Bqt4 and Rap1 proteins in *S. pombe* [86]. In the nineties, studies of telomere localization in plants (i.e., species of beans and peas) using immunofluorescence with labeled specific telomere probes revealed that most of the telomeres localize close to the nuclear periphery [93]. Telomere tethering at the nuclear periphery has also been observed in the worm *Caenorhabditis elegans* (*C. elegans*), which relies on the nuclear protein SUN1 and the telomeric protein POT1 for their anchoring [94]. In *Drosophila*, the telomeres were also observed to be close to the nuclear periphery at one side of the polarized nuclei via high resolution optical microscopy on fixed embryos [95].

**Figure 3:**
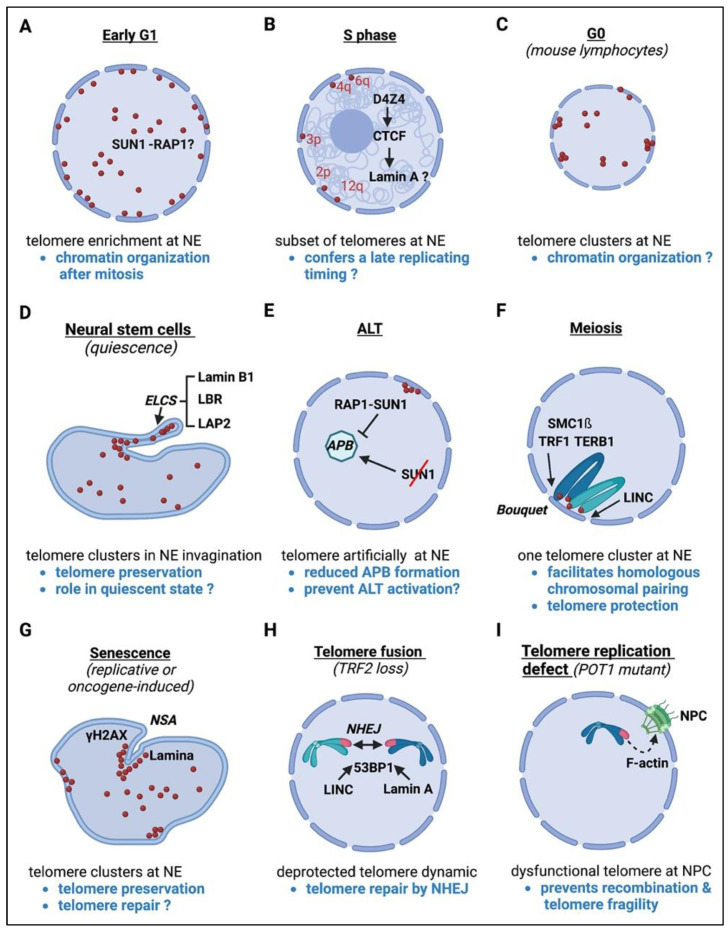
Summary of the different circumstances of mammalian telomere dynamics towards the nuclear envelope (NE), nuclear pore (NPC) and/or lamina. For each case, i.e., cell cycle phases: (**A**–**C**), cellular types: (**D**–**F**), and pathologic events (**G**–**I**), the factors involved, location of telomeres (red dots) and the potential roles of these specific telomere repositionings are indicated. NSA: nuclear shape alterations; ELCS: envelope-limited chromatin sheets; NHEJ: non-homologous end joining; ALT: Alternative Lengthening of Telomeres; APB: ALT-associated PML bodies.

Concerning mammals, a first study, using nuclear fractionation of the interphase nuclei of human cell lines, reported that telomeres are anchored to the nuclear matrix, including NE proteins [96], while, subtelomeric DNA by itself did not anchor to the nuclear matrix. This suggests that, as in yeasts, flies and plants, mammalian telomeres could be attached to the nuclear periphery. However, in contrast to this pioneer study, by using FISH analysis in fixed nuclei and ultrastructure analysis, it was also reported that mammalian telomeres in mouse lymphocytes [97] and human cell lines [98] (i.e., Hela cells) were randomly localized throughout the nucleus, and thereby, a role of the nuclear envelope in telomere maintenance was not expected [98]. In mammalian germ cells, the situation is quite different for meiotic telomeres, which are repositioned at the nuclear periphery during the meiotic prophase, a crucial event for synaptic chromosome pairing and recombination, [99,100,101] (see Section 2.2.6). However, some more recent publications reveal that mammalian telomeres in non-germ cells can also be repositioned at the nuclear rim in a dynamic and transient fashion and under certain circumstances (i.e., senescence, replication, cell cycle phases, cell types), indicating that these relocalizations at the NE are more evolutionary conserved than previously thought and may play significant roles (Figure 3).

### 2.2. Telomere Dynamics in Mammalian Cells

#### 2.2.1. Telomere Relocalization at the NE Periphery during Senescence

In 2008, it was reported that human telomeres form aggregates that are preferentially redistributed to the nuclear periphery, at the lamina, during senescence in mesenchymal stem cells [102] in association with spatial changes in lamina organization (i.e., distortion and intranuclear protuberances). Of note, telomere aggregates were also found colocalized with lamina intranuclear structures in senescent cells. Interestingly, these telomere aggregates colocalized with γH2AX, a biomarker of DNA damage (i.e., DNA double-strand breaks and dysfunctional telomeres) that recruits other DNA damage response factors [57,103]. This suggests that damaged telomeres may be preferentially relocalized at the nuclear lamina during senescence. During oncogene-induced senescence, an enrichment of telomeres located at the nuclear periphery has been observed [104]. Oncogene expression is associated with an increase in telomere replication stress leading to rapid telomere loss and telomere dysfunction [105]. Whether these spatial relocations of telomeres at the nuclear lamina during senescence are a consequence of nuclear lamina reorganization and/or may have a role for damaged telomere preservation or repair is not known.

#### 2.2.2. The Case of Late-Replicating Telomeres

During replication in human cells, a subset of late-replicating telomeres (4q, 2p, 3p, 6q and 12q) are preferentially located at the nuclear periphery [106]. Interestingly, a single repeat of the D4Z4 macrosatellite in the subtelomeric region of a chromosome confers a more peripheral localization and is sufficient to cause the nearby telomere to replicate much later, whereas multiple copies of D4Z4 do not [106,107]. This D4Z4 element has been reported to bind A-type lamins and the CCCTC-binding factor (CTCF), a zing finger transcription factor enriched at the lamina-associated domains that can act either as an activator, repressor or insulator [108]. The perinuclear localization of D4Z4 has also been shown to be dependent on A-type lamins and CTCF, thereby providing a possible tethering mechanism [107,109]. It was previously shown that peripheral localization of other genomic regions, in association with LADs, influences replication timing [110,111]. However, whether these peripheral locations confer a late-replicating phenotype to the associated telomere is not known.

#### 2.2.3. Telomere Repositioning at the Nuclear Periphery in Post-Mitotic and G0 Nuclei

Using time-lapse confocal microscopy, it was shown that a large subset of human telomeres are transitorily repositioned close to the nuclear periphery at the end of mitosis [112] in both primary embryonic and cancer cells (telomerase-positive cells, HeLa), while they are localized throughout the nucleus in the interphase, as observed in previous studies. This transient peripheral localization is initiated during the reformation of the nuclear envelope in late anaphase until early G1, when the NE is fully reconstituted and it concerns approximately half of the telomeres [112]. The mechanisms involved in telomere anchoring is not yet elucidated but may require the SUN1 and RAP1 proteins [112], but it could also rely on different proteins such as lamins and other shelterin proteins than RAP1, since interactions between lamin A and TRF2 [113] and between lamin B1 and both TRF1 and TRF2, have been reported [112,114,115]. During mitosis, the NE breaks down and the NPC disassembles, followed by lamina depolymerization, with the release of lamin A into the nucleoplasm in prophase [116,117], while lamin B1 remains associated in fragmented polymerized fibers with membrane protein aggregates [118,119]. Furthermore, lamin B1 begins to concentrate around chromosomes at the anaphase–telophase transition and assembles into fibers at an early step of the NE reassembly [120], while lamin A is incorporated in lamina much later, in G1. Thus, these lamin B1-associated membrane aggregates could serve as an anchoring point for the telomere during the NE reassembly. Telomere tethering to the NE in post-mitotic cells has been proposed to play a role in the reorganization of chromatin after cell division and/or in telomere maintenance [112]. By using MadID (an optimized approach based on DNA adenine methyltransferase identification (DamID)) [121,122] to identify protein–DNA interactions [123], it has been reported that a small fraction of telomeres are also in contact with the NE during the G1/S phases in HeLa cells, suggesting that a subset of telomeres can also enter into contact with the nuclear envelope outside of mitosis [123].

During the G0 phase of the cell cycle, telomeres tend to form clusters and are mainly located at the nuclear periphery in mouse lymphocytes, while more in the nuclear interior in human lymphocytes [124]. In post-natal quiescent neural stem cells (B1 cells), unusual NE invaginations reminiscent of envelope-limited chromatin sheets (ELCS) have been reported, structures that contain NE proteins, including lamin B1, LBR and LAP2 [125]. These unique nuclear compartments are enriched in telomeres and have specific epigenetic marks of silent chromatin [125]. In budding yeast, telomeres are repositioned in hyperclusters in long-lived quiescent cells. This telomere re-organization has been further involved in the longevity of these quiescent cells [126]. In fission yeast, it has been shown that telomeres are grouped into a single cluster anchored to the NE in quiescent cells via an interaction between the shelterin protein Rap1 and the INM protein Bqt4. This telomere localization is important to preserve their integrity by preventing telomeric rearrangements and repressing TERRA transcription [87]. Thus, this telomere repositioning to the NE may be linked with the quiescence state of the cells and may help to preserve telomeres and genome stability.

#### 2.2.4. Impact of Telomere Tethering to the Nuclear Envelope in ALT Cells

Recently, Teng′s group investigated whether telomere tethering at the NE could affect telomere maintenance of telomerase-negative cancer cells, by the ALT pathway, based on telomere recombination [127]. They show that depletion of SUN1 promotes two characteristics of ALT activity: formation of ALT-associated PML bodies (APB) and C-circles in human ALT cells, suggesting that SUN1 has an inhibitory impact on ALT mechanism. Conversely, artificial tethering of telomeres to the NE by using a RAP1–SUN1 fusion protein, leads to the decrease in APB formation, suggesting that tethering to the NE via SUN1 might inhibit ALT activity. They further show that abolition of the SUN1–RAP1 interaction has no impact on APB formation, indicating the existence of a RAP1-independent pathway for telomere attachment to the NE, that may involve SUN1 [127]. Altogether, these data indicate that NE tethering through SUN1 in ALT cells could have a negative impact on APB formation and, thereby, on ALT recombination mechanism. In line with these data, in budding yeast, it was previously reported that telomere attachment at the NE, which also involves a SUN-domain protein, Mps3, protects telomere tracts from inappropriate recombination [128]. In *C. elegans*, telomere clustering to the NE has been reported in ALT cells, involving SUN1 for telomere anchoring, as in human ALT cells, and the shelterin POT1 [94], indicating a conservation of the anchoring mechanism between yeast, worm and mammals. However, it is not clear whether these telomere relocalizations in clusters at the nuclear periphery are required for the ALT mechanism in *C. elegans*, since some ALT-like strains with random telomere localizations have a C-circle production similar to strains with telomere anchoring to the NE [94]. By contrast, in budding yeast, telomere tethering to the NE in the ALT context has been reported to favor the telomere recombination process. Indeed, after the inactivation of telomerase, senescent cells can establish an ALT mechanism to maintain their telomeres and escape senescence. Interestingly, eroded telomeres relocate to the NPCs, through a mechanism dependent on the small ubiquitin-related modifier (SUMO) pathway, and this repositioning has been reported to promote type II telomere recombination, that is reminiscent of the mammalian ALT mechanism [129]. In addition, surviving cells produce telomeric C-circles, as in human cells, that bind to the NPCs, and this attachment is proposed to promote their formation, suggesting that telomere repositioning at the nuclear periphery in these cells is important for the ALT pathway [130].

#### 2.2.5. Mobility of Dysfunctional Telomeres and the Nuclear Envelope

Relocalization of DNA lesions at the NE has been well described in budding yeast [131,132]. Indeed, in addition to eroded telomeres, persistent DNA double-strand breaks (DSBs), as well as collapsed replication forks, are relocalized to the nuclear pore to be repaired [133,134]. In *Drosophila*, in a similar fashion, DSBs arising in heterochromatic regions are repositioned to the nuclear pore to enable HR repair [135]. Until recently, no such mechanisms of nuclear periphery relocalization of DNA damage were thought to occur in mammalian cells. Artificial tethering of DSBs to the NE (but outside of the NPCs) leads to a delay in the DNA damage signaling response and impairs repair by HR [136], but does not prevent NHEJ nor alternative-NHEJ. However, targeting the DSBs to the nuclear pore does not affect DNA damage signaling nor HR repair [136]. These data suggest that nuclear lamina may confer a roadblock against HR repair to prevent the unwanted recombination at DSBs forming in LADs. This may be linked to the heterochromatic environment of lamina, as a loss of peripheral heterochromatin rescues the recruitment of HR factors [136]. Mammalian dysfunctional telomeres after TRF2 loss have been shown to present a higher mobility than undamaged telomeres by time-lapse microscopy in mouse embryonic cells [137]. This dynamic was shown to depend on ATM kinase, and 53BP1, which is further involved in promoting NHEJ at dysfunctional telomeres [137]. It was later shown that this 53BP1-depedent dynamic of dysfunctional telomeres requires components of the cytoskeleton, the microtubules and the LINC complex (i.e., SUN1, SUN2 and nesprin-4) [138]. However, no clear peripheral localization of these dysfunctional telomeres was observed. In addition, a role of lamin A in the repair of dysfunctional telomeres has been proposed. Indeed, in mouse embryonic fibroblasts (MEF), lamin A deficiency decreases the number of telomeric fusions, normally induced by overexpression of the dominant negative form of TRF2 (TRF2^ΔBΔM^) [139]. This suggests a role for lamin A in promoting the processing of dysfunctional telomeres by NHEJ, that may be linked to its role in stabilizing the 53BP1 protein [140]. Recently, the first demonstration of the relocalization of mammalian dysfunctional telomeres at the nuclear periphery was reported [141]. In this study, telomeres rendered dysfunctional through mutations of the shelterin POT1 that induces telomere replication defects (i.e., mutations located in the OB-fold domain involved in the single-stranded telomeric binding of POT1 to DNA), lead to a relocalization of damaged telomeres at the nuclear pore via an F-actin-dependent mechanism. The authors further showed that this relocalization to the NPC prevents inappropriate recombination between telomeres of sister chromatids and also telomere fragility [141], a mark of telomere replication stress [142,143]. These data unveil a role of the NPC in the maintenance of mammalian telomeres, especially in the resolution of telomeric replication defects to ensure telomere stability [141], that has been evolutionary conserved from yeast to mammals.

#### 2.2.6. The Case of Meiotic Cells

The meiotic telomere repositioning at the nuclear periphery during meiosis has been conserved through the evolution from yeast to human and involved conserved SUN and KASH proteins [86,88]. During mammalian meiosis, important changes in chromatin organization occur in early prophase I, leading to the formation of the so-called “bouquet”, a polarized chromosomal configuration, involving telomere attachment to the NE followed by chromosomal pairing and homologous recombination and the subsequent formation of haploid gametes [144]. Indeed, to form the “bouquet” at the leptotene/zygotene transition, telomeres are repositioned at the nuclear periphery and move along the NE to form a cluster at one side of the nucleus, facilitating the transient configuration of the bouquet of the meiotic chromosomes [88]. The movement of telomeres is important to facilitate the search for chromosome homology and the further alignment and pairing of chromosomes in pachytene [88,145]. Disruption of telomere anchoring to the NE during meiosis leads to severe defects in the homologous chromosomal pairing, synapsis and recombination [146,147], thereby showing that telomere tethering to the NE during meiosis is essential for the progression of mammalian meiosis. The proteins SUN1 and SUN2 [146,147] are essential for the anchoring of meiotic telomeres to the NE. In addition to SUN proteins, several factors involved in the NE tethering of telomeres have been identified [148]. Among them, TERB1, a meiosis-specific TRF1-binding protein involved in the connection of SUN proteins to telomeres, and the KASH protein KASH5 involved in telomere–NE anchoring, have been shown to play important roles in the movement of telomeres during meiosis, and their defects strongly affect homologous pairing [149,150,151]. In contrast with yeast that requires the RAP1 ortholog for meiotic telomere anchoring, mammalian meiotic cells do not require the RAP1 protein for their attachment to the NE [101,152]. A meiosis-specific cohesin, SMC1β, has been reported to be required for complete perinuclear telomere rearrangement [153] and to be involved in meiotic telomere protection [154]. More recently, the factor FBXO47, expressed specifically in meiosis, has been reported to contribute to shelterin stabilization at meiotic telomeres [155]. In addition, the shelterin TRF1, together with Speedy A and Cdk2, plays a role in protecting telomeres during the bouquet stage [156], as well as in telomere attachment to the NE through its interaction with TERB1 [157]. The lamin C2, a short isoform of lamin A expressed specifically in meiotic cells, has been also involved in telomere repositioning during meiosis, and its depletion leads to severe meiosis defects [158].

All these data show that mammalian telomeres are highly dynamic entities during the cell cycle and in a more close interaction with the NE than previously thought. The different circumstances of telomere relocalization are summarized in Figure 3.

## 3. Focus on Lamina and Telomere Maintenance

### 3.1. Lamins and Telomeric Sequences

More than thirty years ago, lamins were shown to present DNA-binding properties in vitro, especially for G-rich DNA sequences, with lamin A/C exhibiting higher affinity for human telomeric repeat sequences [159,160,161]. Using chromatin immuno-precipitation, it was later shown that telomere sequences are specifically bound by lamin A- and B-types and it was confirmed that lamin A appears to have more affinity than lamin B for telomere sequences [102]. In accordance with these findings, a subset of telomeric regions was found in the LAD regions of human cells [104].

### 3.2. Lamina and Shelterin

Links between mammalian telomeric protein complex, shelterin, and the nuclear matrix were first reported by the group Titia de Lange, more than twenty-five years ago in HeLa cells [98]. Indeed, the shelterin TRF1 was shown to co-fractionate with the inner nuclear matrix, while no evidence of interaction between telomeric proteins and nuclear shells (i.e., peripheral structures of the nuclear matrix) were found by co-fractionation, in accordance with the localization of the telomeric proteins throughout the nucleus and not preferentially enriched at the nuclear periphery. Of note, mammalian lamins are found in both the inner nuclear matrix and at the nuclear periphery [8,10,162]. In the frog, the shelterin protein TRF2, but not TRF1, was reported to colocalize with the NE in the oocyte [163], and with NE remaining fragments marked with lamin B in mitotic mouse cells [164]. It was proposed that TRF2 could be the protein involved in telomere anchoring to the NE in somatic cells [164]. Interestingly, TRF2 displays some sequence homologies with the Rod domain motifs of intermediate filaments found in lamin protein sequences [164]. These homologies were reported to be limited in the dimerization domain of TRF2 and higher in its linker region (also known as the Hinge region [61]) involved in protein–protein interactions including the interacting site with RAP1 [114,164,165]. The first evidence of an interaction between shelterin and lamins was reported for the shelterin TRF1 and lamin B1, but not in endogenous conditions [112]. Indeed, lamin B1 was co-precipitated with TRF1 in a sample from enriched post-mitotic cells overexpressing EGFP-TRF1 [112]. Two years later, a small fraction of TRF2 was reported to be associated with lamin A [113], although whether this interaction is direct is not known. This interaction between lamin A is proposed to participate in the formation of loops between terminal telomere sequences and interstitial telomere sequences (ITS), termed interstitial telomeric loops (ITLs). These ITLs are suggested to play a role in telomere protection and/or stabilization [113,166]. It was recently reported that the linker region of TRF2 may be involved in lamin A-TRF2 association [167]. However, a previous study reported that the dominant-negative form of TRF2 (TRF2^ΔBΔM^), which can no longer bind telomere [113], but still contains the linker region, lacks an interaction with lamin A, suggesting that lamin A is preferentially associated with DNA-bound TRF2. Of note, progerin, the aberrant form of lamin A, depleted of 50 amino acids in its C-terminal part and responsible for Progeria disease, lacks an interaction with TRF2 [113]. Recently, our team showed that lamin B1 interacts with TRF2 and its binding partner RAP1 [115]. Our data further suggest that this interaction involves the head-coil 1 domain of lamin B1, rich in coiled-coil motifs involved in protein oligomerization and protein–protein interaction, and the hinge domain of TRF2, which contains a RAP1 binding site [115]. Since an increase in TRF2-lamin B1 interaction upon lamin B1-overexpression is associated with a mislocalization of TRF2 at the NE, and increased telomere dysfunction (i.e., telomere losses and fusions) [115], we propose that this interaction may be involved in telomere maintenance. Whether lamin B1, through its interaction with the shelterin complex, is also involved in transient relocalizations of telomeres at the nuclear periphery, such as those observed in early G1, or for late-replicative telomeres (see Section 2.2.2), or in the stabilization of ITLs, as proposed for lamin A, remains unknown.

### 3.3. Other Lamina-Interacting Proteins and Telomere Maintenance

Besides lamins, additional proteins linking telomere homeostasis and lamina have been previously identified (Table 1). Among them, AKTIP, which is an interacting factor of lamin A and B1 and enriched at the nuclear rim [19], has been shown to directly interact with the shelterin subunits TRF2 and TRF1 [168]. Depletion of AKTIP was reported to induce TIFs, sister telomere associations and the telomere fragility phenotype [168], suggesting a requirement of AKTIP for telomere maintenance. AKTIP was further proposed to be required for telomere replication in cooperation with TRF1 [168].

LAP2α, another nucleoskeletal protein that interacts with lamin A/C [173] particularly in the G1 phase, associates with telomeres, forming core structures during the early phases of nuclear reassembly. This association is proposed to play a role in the tethering of telomeres during nuclear reformation to favor chromatin reorganization [169]. Of note, BAF, which is an interacting factor of LAP2α, relocalizes to these LAP2α core structures, suggesting that BAF may also be involved in chromatin reorganization during nuclear reassembly [169]. Of note, LAP1, another LAP protein, has also been reported to be associated with the shelterin protein TRF2 [171] and a role of this interaction in the DNA damage response has been proposed. Recently, the INM protein LBR has been also linked with telomeres. Indeed, an association between LBR and the shelterin TRF2 has been reported, but not with TRF1 [172], and depletion of LBR leads to an upregulation of TRF1 together with an induction of chromosomal instability (i.e., chromosomal losses and translocations) in colorectal cancer cells. Co-depletion of LBR and TRF1 prevents chromosomal instability, suggesting that LBR, through its links with shelterin, may be involved in chromosomal stability [172]. All these data indicate that lamins and lamin-associated factors are emerging players in mammalian telomere maintenance that contribute to telomere stability.

## 4. Nuclear Envelope Alterations and Telomere Dysfunction

### 4.1. Laminopathies and Telomere Dysfunction

Links between lamina alterations and telomere dysfunction were first reported in the rare premature aging disease, Hutchinson–Gilford Progeria (HGPS) [174], caused by an abnormal truncated form of lamin A, called progerin [175] (Table 2). Indeed, cells from HGPS patients or cells artificially expressing progerin exhibit decreased telomere length [174,176,177], along with other cellular phenotypes including premature senescence, nuclear shape alterations (NSA) and persistent DNA damage [178]. In progerin-expressing cells, the formation of TIFs, telomere aggregates and telomeric aberrations (i.e., telomere fusions, telomere losses and telomere doublets) have also been reported [179]. Mouse cells lacking lamin A also exhibit shorter telomeres, a change in nuclear distribution of telomeres toward the nuclear envelope, alteration of the telomeric chromatin structure in association with an increase in genomic instability [139]. Recently, in human cells, progerin has been shown to induce a genome-wide defect in replication timing [180], telomeric replication stress marked by telomere fragility (or multi-telomere signals at one chromatid end), and replication forks’ perturbation at telomeres, in addition to the alteration of telomere organization and changes in heterochromatin status [181]. Progerin expression induces a telomere replication timing defect and, interestingly, telomere fragilty can be rescued by dNTP [181]. Although LAP2α interacts with lamin A in normal cells, in HGPS cells, progerin fails to interact properly with LAP2α and the association of LAP2α with the telomere is compromised [170,173]. Increasing the level of LAP2α protein in HGPS cells rescues their proliferation defects, the loss of the heterochromatic marks H3K9me3, premature senescence, and DNA damage induction. By contrast, depletion of LAP2α enhances the proliferation defects of HGPS. cells. The authors suggest that impaired LAP2α association with telomeres participates in several progerin-induced cellular defects.

It is noteworthly that the proliferative defects observed in HGPS cells or in cells ectopically expressing progerin can be compensated by the expression of hTERT, the catalytic subunit of telomerase [179,190]. In addition, inhibition of the telomere DNA damage response has been reported to restore some deleterious phenotypes of HGPS syndrome [191]. All these data indicate that telomere dysfunction contributes to the pathogenesis of this lamin-related disease, and conversely, lamina alterations directly impact telomere function and/or homeostasis. 

### 4.2. Lamin B1 Dysregulation and Telomere Alterations

Lamin B1 overexpression leads to NSA as well as proliferation defects and induction of premature senescence [186,192]. It was shown that the expression of the telomerase catalytic subunit hTERT can rescue the proliferative defects induced by lamin B1 increase, suggesting that telomere dysfunction could be at the origin of this phenotype [192]. In accordance with this statement, we showed that lamin B1 overexpression induces telomere aberrations (i.e., telomere losses and telomere fusions) linked to a mislocalization of the shelterin proteins TRF2 and RAP1 to the nuclear envelope periphery [115].

A duplication of the lamin B1 gene (*LMNB1*) is involved in the rare adult-onset neurodegenerative pathology, autosomal dominant leukodystrophy (ADLD) [182]. Defects of the autonomic nervous system are one of the main symptoms of this pathology and are thought to be caused by demyelination of the central nervous system that appears in an age-dependent manner [193]. The increase in lamin B1 has been involved in the demyelination phenotype via a downregulation of genes involved in myelin synthesis in oligodendrocytes [194]. Many pathologies of the central nervous system, as well as brain tumors, have been associated with damage to telomeres or telomerase [195]. To date, the existence of telomeric defects associated with ADLD pathology has not been reported. One can propose that a telomere dysfunction in patient cells, such as an impact on telomere size, could be involved in the appearance of the age-dependent phenotype of ADLD.

In addition, an increase in lamin B1 together with NSA has been observed in the neurodegenerative disorder with a premature aging component, Ataxia-Telangectasia (A-T) due to mutation in the *ATM* gene, and in the premature aging syndrome, Werner syndrome, involving mutation in the *WRN* gene [186,187]

. It was further shown that the elevated level of lamin B1 accounts for nuclear shape alterations and senescence in A-T cells [186]. Interestingly, in both disorders, cells from patients exhibit telomere defects. The kinase ATM, as well as the WRN helicase, are involved in telomere maintenance [78,79,188,189,196,197], and their alterations are directly linked with the increased telomere dysfunction associated with these pathologies. As lamin B1 overexpression is involved in telomere instability, it might also contribute to the telomeric phenotype, in addition to the premature senescence and NSA phenotypes, in cells from patients suffering from these diseases associated with premature aging.

### 4.3. NSA, Telomeres and Cancer

NSA is a hallmark of cancer cells and lamins have been shown to be deregulated in several cancer types and their levels of dysregulation have been correlated with tumor aggressiveness in different types of cancer [3]. Telomere dysfunction could be a driver of genome instability and promote tumor progression [198]. Interestingly, the telomere state and lamin A amount have been identified as factors that can modulate the cellular sensibility of cancer cells in response to a telomerase inhibitor treatment [199]. Indeed, short telomeres and a low level of lamin A in cancer cells confer a higher sensitivity to the deleterious effect of this anti-cancer drug. As we previously discussed above, lamin dysfunctions have been reported to impact telomere maintenance. Altogether, these studies indicate that both the telomere and lamin systems are functionally linked and the maintenance of their states is essential for genome stability. In addition, other proteins of the nuclear envelope have been shown to induce telomere damage, such as AKTIP and LBR (see Section 3.3), which have been associated with tumorigenesis [172,200,201]. Thereby, more generally, nuclear envelope dysfunction may have an impact on telomere maintenance in cancer cells.

## 5. Conclusions

Over the last few years, studies in mammalian somatic and cancer cells have revealed that telomere connections to the nuclear envelope are more evolutionary conserved than previously thought. Apart from the case of meiotic telomeres, for which their relocalizations in cluster at the nuclear periphery are essential for chromosome pairing from yeast to human, mammalian telomere repositioning at the nuclear periphery has been shown to occur transiently at specific moments of the cell cycle and to be involved in different cellular processes, such as telomere replication timing, repair, chromatin organization and gene expression. Taken together, all these studies indicate that the nuclear envelope confers a special compartment to telomeres that plays an important role in preserving their integrity, and beyond, the genome stability. Recent advances have also shown that lamins, key components of the nuclear envelope, are involved in telomere maintenance, which highlight the importance of nuclear envelope integrity in preserving telomere stability and preventing further genomic instability, the source of tumorigenesis.

## Figures and Tables

**Table 1:** Summary of associations between lamins and lamina-associated proteins and telomeric proteins, impact of their alterations on telomeric phenotypes, and their potent functions at the mammalian telomere.

Protein	Telomeric Associations	Defect > Telomeric Phenotypes ^1^	Telomere Function ^1^	Studies
Lamin A/C	TRF2	Loss > Telomere Shortening Reduced ITL formation	ITLs formation Telomere maintenance	[113,139]
Lamin B1	TRF2 RAP1	Overexpression > TRF2/RAP1 mislocalization at the NE TIFs, Telomere loss and fusions	Shelterin stability Telomere maintenance	[115]
AKTIP	TRF1 TRF2	Depletion > TIFs Sister telomere fusions Telomere fragility	Telomere maintenance Telomere replication	[19,168]
LAP2α	Partially localized with TRF1 Enriched at telomere during anaphase	-	Surrounding telomere: Telomere stabilization? Telomere repositioning during NE reassembly?	[169,170]
LAP1	TRF2 (outside telomere)	-	-	[171]
LBR	TRF2	Loss > Upregulation of TRF1 Chromosomal instability	-	[172]

^1^ The dash indicates “not studied”.

**Table 2:** Lamin-associated diseases, nuclear shape alterations (NSA), and telomere dysfunction (TD).

Lamin-Related Diseases	Features	Gene/ Protein Involved	NSA	TD	References
HGPS ^1^	Premature aging	*LMNA* or *ZMPSTE24*/ Progerin (aberrant truncated lamin A)	yes	TIFs Telomere shortening Telomere fusions Telomere fragility	[174,175,176,177,179,181]
ADLD ^2^	Age-dependent neurodegenerative disease	*LMNB1*/ Lamin B1 increase	yes	-	[182]
Ataxia-Telangiectasia	Premature aging Neurodegenerative disease	*ATM*/ATM deficiency and lamin B1 increase	yes	Telomere shortening Telomere fusions	[183,184,185,186]
Werner syndrome	Premature aging Cancer predisposition	*WRN*/WRN deficiency and lamin B1 increase	yes	Telomere shortening Defective telomere lagging-strand synthesis	[187,188,189]

^1^ HGPS: Hutchinson–Gilford Progeria Syndrome; ^2^ ADLD: Autosomal Dominant Leukodystrophy.

## Data Availability

Not applicable.
